# Identification of Plasmid-Encoded sRNAs in a *bla*_NDM-1_-Harboring Multidrug-Resistance Plasmid pNDM-HK in *Enterobacteriaceae*

**DOI:** 10.3389/fmicb.2018.00532

**Published:** 2018-03-27

**Authors:** Hoi-Kuan Kong, Xuan Liu, Wai U. Lo, Qing Pan, Carmen O. K. Law, Ting F. Chan, Pak L. Ho, Terrence C. K. Lau

**Affiliations:** ^1^Department of Biomedical Sciences, City University of Hong Kong, Kowloon, Hong Kong; ^2^Department of Microbiology and Carol Yu Centre for Infection, The University of Hong Kong, Pokfulam, Hong Kong; ^3^School of Life Sciences, The Chinese University of Hong Kong, Hong Kong, Hong Kong

**Keywords:** sRNA, pNDM-HK, plasmid, NDM-1, multidrug-resistance, phylogenetic analysis

## Abstract

Small RNAs (sRNAs) play significant roles in regulating gene expression post-transcriptionally in response to environmental changes in bacteria. In this work, we identified and characterized six novel sRNAs from an emerging multidrug-resistance (MDR) plasmid pNDM-HK, a New Delhi metallo-β-lactamase 1 gene (*bla*_NDM−1_)-carrying IncL/M plasmid that has caused worldwide threat in recent years. These sRNAs are located at different regions of pNDM-HK, such as replication, stability, and variable regions. Moreover, one of the plasmid-encoded sRNAs (NDM-sR3) functions in an Hfq-dependent manner and possibly plays roles in the fitness of pNDM-HK carrying bacteria. In addition, we attempted to construct the phylogenetic tree based on these novel sRNAs and surprisingly, the sRNA-phylogenetic tree provided significant information about the evolutionary pathway of pNDM-HK, including possible gene acquisition and insertion from relevant plasmids. Moreover, the sRNA-phylogenetic tree can specifically cluster the IncM2 type and distinguish it from other IncL/M subtypes. In summary, this is the first study to systematically identify and characterize sRNAs from clinically-isolated MDR plasmids. We believe that these newly found sRNAs could lead to further understanding and new directions to study the evolution and dissemination of the clinically MDR bacterial plasmids.

## Introduction

Small regulatory RNAs (sRNAs) control transcriptional and post-transcriptional gene expression in prokaryotes. They are usually 50–250 nucleotides (nt) in length and can be classified either as *cis*-encoded or *trans*-encoded (Massé et al., 2003; Waters and Storz, 2009; Gottesman and Storz, 2011; Storz et al., 2011). *Cis*-encoded sRNAs are expressed from the opposite strand of target mRNA, usually perfectly complementary to their targets. The high complementarity leads to gene expression modulation through RNA degradation and transcription termination (Wagner et al., 2002; Brantl, 2007; Georg et al., 2009; Güell et al., 2009; Toledo-Arana et al., 2009). *Trans*-encoded sRNAs are transcribed from independent loci of their target mRNAs and possess limited complementarity to their targets (Gottesman, 2005; Aiba, 2007; Prévost et al., 2007; Waters and Storz, 2009). Most sRNAs reduce translation efficiency through base pairing to the 5′-untranslated region (5′-UTR) (Massé et al., 2003; Waters and Storz, 2009; Gottesman and Storz, 2011; Storz et al., 2011); while certain sRNAs relieve the secondary structures around ribosome binding sites, resulting in gene activation (Fröhlich and Vogel, 2009). Regulation by sRNA-mRNA base pairing has shown to be more advantageous in providing a quick response compared with protein-DNA and protein-protein interactions (Storz et al., 2004; Brantl, 2007; Shimoni et al., 2007). In recent years, it has been proven that sRNAs are critical in physiological responses including oxidative and periplasmic stress, osmotic shock, iron limitation, glucose limitation, and accumulation in gram-negative bacteria (Altuvia et al., 1997; Masse and Gottesman, 2002; Vanderpool and Gottesman, 2004; Wilderman et al., 2004; Majdalani et al., 2005; Gottesman et al., 2006; Guillier and Gottesman, 2006). Various gram-positive bacteria also use sRNAs to regulate genes associated with quorum sensing and virulence factors, revealing the importance of sRNAs in pathogenesis (Kreikemeyer et al., 2001; Mangold et al., 2004; Pallen and Matzke, 2006; Boisset et al., 2007). However, most of the identified sRNAs are chromosomally encoded, and only a few are plasmid-encoded sRNAs. The first plasmid-encoded sRNA, RNAI, was identified from the *Escherichia coli* plasmids, ColE1 (Tomizawa, 1986; Tomizawa et al., 2001) and R1 (Stougaard and Nordström, 1981), in 1981. This *cis*-encoded antisense RNA regulates plasmid replication and controls the plasmid copy number. Most of these known plasmid-encoded sRNAs function through modulating plasmid replication, post-segregational stability and conjugation frequency (Kumar and Novick, 1985; Eisen et al., 2000; Weaver, 2007; Brantl, 2012).

Over the last decade, the extended-spectrum β-lactamases (ESBLs), which hydrolyses extended-spectrum cephalosporins, are spreading among *Enterobacteriaceae* through mobile elements, such as conjugative plasmids (Ho et al., 2012a,b, 2015). For bacteria expressing β-lactamases genes (such as *bla*_SHV_, *bla*_TEM_, and *bla*_CTX−M_), carbapenems are regarded as the only class of agents for treatment. However, carbapenem treatment loses its clinical value due to a plasmid-harbouring novel resistance gene, New Delhi metallo-β-lactamase 1 gene (*bla*_NDM−1_), which was firstly identified in 2009 in India and the UK (Kumarasamy et al., 2010). Ever since its discovery, the NDM-1-carrying gram-negative *Enterobacteriaceae* has become pandemic. One typical NDM-1-carrying plasmid is pNDM-HK, which was first isolated in an *E. coli* strain from Hong Kong in October 2009. Plasmid pNDM-HK is a 90-kb plasmid comprised of a 55-kb backbone and a 28.9-kb variable region (Ho et al., 2011). It belongs to the IncL/M family, one commonly known to disseminate multidrug-resistance (MDR) genes (Carattoli, 2009). The pNDM-HK plasmid has been proposed to evolve through complex pathways via sequential acquisition of MDR genes (Bonnin et al., 2013). The backbone of pNDM-HK shares 97% similarity with a plant pathogen *Erwinia amylovora*-hosting plasmid pEL60. The variable region has a composite transposon-like structure that encodes intact or truncated genes associated with resistance to β-lactams (*bla*_NDM−1_, *bla*_TEM−1_,*bla*_DHA−1_), aminoglycosides (*aacC2, armA*), macrolides (*mel, mph2*), and sulfonamides (*sul1*). This plasmid possesses high homology to pCTX-M3, which has contributed to the dissemination of CTX-M type β-lactam resistance except in the presence of the *bla*_NDM−1_ gene (Novais et al., 2010).

Identification and characterization of bacterial genomes are essential for treatment and disease control. Besides whole-genome sequencing, RNA-Seq is another powerful technique to characterize the physiology of bacteria. RNA-Seq enables a comprehensive overview of gene expression at different stages of pathogenic conditions, providing a better understanding of the survival mechanisms, drug resistance profiles as well as infection strategies of pathogens (Sorek and Cossart, 2010; Westermann et al., 2012; Deurenberg et al., 2017). In the last decade, most genomic studies focused on the evolution, dissemination, and gene acquisition of clinically-isolated MDR plasmids but little is known about the plasmid-encoded sRNAs. Therefore, in this study, we set out to identify and characterize sRNAs encoded from pNDM-HK plasmid. Six pNDM-HK encoded sRNAs were distributed within replication, stability, and variable regions of pNDM-HK plasmid. A phylogenetic tree constructed by these sRNAs revealed important information on the evolutionary process of pNDM-HK including possible gene acquisition and insertion from relevant plasmids. Surprisingly, the sRNA-phylogenetic tree is able to cluster and distinguish IncM2 types from other IncL/M plasmids, suggesting a novel approach to constructing the phylogenetic tree without the need for whole-plasmid sequencing. To further understand the role of these plasmid-encoded sRNAs, we examined NDM-sR3 which is located in the variable region, and found that NDM-sR3 regulates genes related to plasmid fitness in an Hfq-dependent manner. This is the first systematic analysis of sRNAs in clinically-isolated MDR plasmids, and we believe that our phylogenetic and functional studies of these plasmid-encoded sRNAs will help to unveil the mechanisms in the evolution and dissemination of MDR plasmids.

## Materials and methods

### Bacterial strains and growth conditions

*E. coli* strains and plasmids used in this study are listed in Table S1. DH5α and BL21(DE3)pLysS were utilized for cloning and overexpression of Hfq proteins, respectively. Transconjugant *E. coli* J53 harboring pNDM-HK was a laboratory stock from PL Ho's (Ho et al., 2011). Wild-type *E. coli* strain MG1655 was adopted for assays and phenotypic studies. Bacteria were grown in LB broth at 37°C under shaking at 250 rpm to the phases indicated. Antibiotic concentrations in growth media were applied as below: ampicillin 100 μg/ml, kanamycin 20 μg/ml, or chloramphenicol 25 μg/ml.

### Plasmid and strain construction

Plasmid preparation, DNA purification, restriction endonuclease cleavage, ligation, and transformation followed protocols of kits or standard methods. The in-frame knockout of *hfq* in MG1655 followed the methods using IPTG-induced recombinase from pKM208 and electroporation (Murphy and Campellone, 2003). The transcription unit (TU) of NDM-sR3 (sR3) was amplified from the plasmid DNA of pNDM-HK employing primers XhoI-sR3-F and XhoI-sR3-R, and inserted into the *Xho* I site of pTL01, a derivative of pACYC184 carrying an additional *Xho* I restriction site, generating pTL02.

### RNA extraction and sRNA isolation

*E. coli* cell pellets were re-suspended in extraction buffer (10 mM Tris pH 8.0 and 1 mM EDTA) and incubated with 20 mg/ml lysozyme (Sigma) for 5 min at room temperature. The mixtures were then mixed in three volumes of TRIzol reagent (Invitrogen) and RNA was extracted by adding one volume of chloroform followed by centrifugation. Total RNA was precipitated in isopropanol and its quality and quantity were determined with a NanoDrop ND-1000 spectrophotometer (Thermo) and TAE agarose gel electrophoresis.

Small RNA (sRNA) was separated and enriched from total RNA by utilizing the mirVana™ miRNA Isolation Kit (Life Technologies) and subjected to the MICROBExpress Kit (Ambion) and Ribo-Zero rRNA Removal Kit (Epicenter) to eliminate rRNA according to the manufacturer's instructions. The concentration of sRNAs was also confirmed by ND-1000 (Thermo), and its quality and integrity were then monitored by Bioanalyzer (Agilent) using RNA 6000 Pico Kit (Life Technologies).

### Library construction and sRNA sequencing

The rRNA-depleted RNA was used to construct the library with the Ion Total RNA-Seq Kit v2 (Ambion) according to the manufacturer's protocol. Libraries were next sequenced using the Ion Torrent Sequencing platform on Ion 316 Chips (Life Technologies). Reads were mapped to reference genome *E. coli* str. K-12 substr. MG1655 (GenBank accession NC_000913.3) and plasmid pNDM-HK (GenBank accession: NC_019063.1) using TMAP (Smith and Waterman, 1981; Ning et al., 2001; Li and Durbin, 2009, 2010; Li, 2012) and only reads with high mapping quality were kept for downstream analysis. Mapping quality was defined as the rate of uniquely mapped reads. The unique mapping rate of *E. coli* J53 and J53 carrying pNDM-HK were 88 and 98%, respectively. The mapped sequencing reads were visualized by Integrated Genome Viewer (IGV) 2.3.34 (Robinson et al., 2011). Small RNAs were searched in antisense and intergenic regions based on read-mapping patterns. The initial and terminal bases of sRNAs were determined by choosing nucleotides that had more than 10% coverage of its maximum reads. The sequencing data was deposited in the NCBI Sequence Read Archive (SRA) database (accession number: SRR6703077).

### 5′-rapid amplification of cDNA ends (5′RACE)

5′-Rapid amplification of cDNA ends (5′RACE) was performed with FirstChoice RLM-RACE Kit (Life Technologies) according to the manufacturer's instructions. Briefly, total RNA was reverse transcribed into cDNA by M-MLV Reverse Transcriptase. PCR was first performed with 5′ RACE Outer Primer (Life Technologies) and a gene-specific outer primer. A nested PCR was next performed to enhance the specificity with 5′ RACE Inner Primer (Life Technologies) and gene-specific inner primer. The round two PCR products were excised and ligated with pGEM-T Easy vector (Promega) and transformed into DH5α. Single colonies were selected and sequenced with M13 forward primer.

### Northern blot analysis

Ten μg total RNA was separated on a 6% polyacrylamide gel containing 8 M urea and transferred onto a Hybond-N nylon membrane (GE Healthcare) at 150 mA for 1 h. RNA was cross-linked with the membrane under UV for 2 min. The blot was then pre-hybridized with 5 ml Ultrasensitive Hybridization buffer (Ambion) at 42°C for 1 h. Oligonucleotide probes 5′-end labeled with [γ-^32^P]-ATP (PerkinElmer) using T4 polynucleotide kinase (NEB) were added for blot hybridization at 42°C overnight. For NDM-sR6, the ^32^P-labeled RNA probe was synthesized by *in vitro* transcription using MEGAscript T7 Transcription Kit (Ambion) with addition of [α-^32^P]-UTP (PerkinElmer). The blot was washed twice with 20 ml SSC buffer for 10 min, and an image was obtained by phosphor-imager. *E. coli* 5S rRNA was probed as the loading control.

### RNA half-life determination

*E. coli* MG1655 and MG1655Δ*hfq* harboring pTL02 were grown to exponential phase OD_600_~0.6. Cells were treated with 250 μg/ml rifampicin to terminate RNA biosynthesis. Cultures were, respectively, harvested at time points 0, 1, 2, 5, and 10 min after addition of rifampicin. Total RNA of these samples was extracted and NDM-sR3 level was examined by Northern blot. The RNA levels over time were calculated as the percentages to that at time point zero. The *in vivo* degradation curve was calculated following the fitted equation *N*(*t*) = *N*(0) ∗ 2^−*t*/*t*1/2^, where *N*(*t*) is the RNA level at specified time point, *t, N*(0) is the initial RNA level, and *t*_1/2_ is the half-life.

### Purification of Hfq

The open-reading frame of *E. coli hfq* was amplified from MG1655 and cloned into pET28a to generate pET28a-hfq with a C-terminal 6 × His tag, and this plasmid was transformed into *E. coli* BL21(DE3)pLysS for protein purification. Cells containing pET28a-hfq were grown to OD_600_ ~0.6 at 37°C, and Hfq protein was induced by adding 2 mM IPTG. After shaking for 4 h, cells were harvested by centrifugation and resuspended in lysis buffer (20 mM Tris-HCl, 150 mM NaCl, 0.5% Triton X-100, 0.4 mM PMSF, and 2 mM ß-mercaptoethanol, pH 7.5). Then, bacteria were lysed by sonication and precipitate was removed by centrifugation. Supernatant was passed over TALON Cobalt column (Clontech), followed by washing with five bed volumes of high salt buffer (20 mM Tris-HCl, 500 mM NaCl, 20 mM imidazole, 2 mM ß-mercaptoethanol, pH 7.5) and low salt buffer (20 mM Tris-HCl, 150 mM NaCl, 20 mM imidazole and 2 mM ß-mercaptoethanol, pH 7.5). Hfq protein was eluted by elution buffer (20 mM Tris-HCl, 150 mM NaCl, 250 mM imidazole, and 2 mM ß-mercaptoethanol, pH 7.5). Purified Hfq was dialyzed against storage buffer (20 mM Tris-HCl, 200 mM NaCl and 10% glycerol, pH 7.5) at 4°C overnight. Hfq purity and concentration were determined by A_280_/A_260_ and SDS-PAGE (purity > 95%).

### Electrophoretic mobility shift assay

[α-^32^P]-UTP labeled NDM-sR3 RNA (0.4 pmol) and indicated amounts of purified His-Hfq_6_ were mixed in 20 μl of binding buffer (10 mM Tris-HCl, 0.1 mM EDTA, 10 mM NH_4_Cl, 10 mM NaCl, 10 mM KCl, pH 7.5) containing 1 U RNasin (Promega). The mixtures were incubated for 30 min at 37°C and stopped by adding 4 μl loading buffer (25% glycerol, 0.05% bromophenol blue). The samples were analysed on an 8% native polyacrylamide gel containing 5% glycerol. The gel was subjected to autoradiography after electrophoresis.

### Quantitative real-time PCR (qRT-PCR)

The total RNA for qRT-PCR was treated with 2U TURBO DNase (Ambion) at 37°C for 30 min twice, and incubated with 1/10 (v/v) inactivation reagent (Ambion) to inactivate DNase. The recovered RNA was subjected to DNA contamination testing by PCR using *gapA* primers. One μg qualified RNA was next reverse transcribed into cDNA by the Superscript III First-Strand Synthesis System (Life Technologies) following the instructions of the manufacturer. Primers for qRT-PCR were designed with Primer3 software and are listed in Table S2. The gene, *gapA*, served as the endogenous control for normalization of target genes. qRT-PCR was set up with 5 μl Power SYBR Green PCR Master Mix (Life Technologies), 1 μl cDNA, 2 μM forward and reverse primers, and appropriate nuclease-free water to a total of 10 μl per reaction. The PCR was run on a 7500 Fast Real-time PCR System (ABI) with a program for 95°C 5 min for 1 cycle, and 95°C 15 s, 60°C 1 min for 35 cycles. The Ct-values from all qRT-PCR reactions in triplicate were analyzed to detect target gene expressions.

### Phylogenetic analysis

Small RNA or pNDM-HK sequences were taken as input queries and submitted to the Blast (Altschul et al., 1997; Tatusova and Madden, 1999; Brilli et al., 2008) website to search for plasmids that contained similar input sequences under stringent criteria (megablast). Multiple sRNAs were concatenated into one long query sequence. ClustalW was applied to generate multiple alignments and phylogenetic trees (Thompson et al., 1994; Chenna, 2003; Larkin et al., 2007). Only high coverage and identity (at least 70%) alignments were kept for phylogenetic tree construction employing the neighbor-joining method. Max sequence difference was set at 0.1. The resulting phylogenetic tree was plotted with MEGA6 (Tamura et al., 2013).

## Results

### Small RNA sequencing and identification of pNDM-HK-encoded sRNAs

We previously sequenced the pNDM-HK plasmid isolated from an MDR *E. coli* strain in Hong Kong. The plasmid belonged to the broad host range IncL/M incompatibility group. In order to identify novel sRNAs encoded in this plasmid, we performed sRNA sequencing of the pNDM-HK transconjugant. The pNDM-HK plasmid was conjugated into *E. coli* K-12 J53 (Yi et al., 2012) and the transconjugant was selected and isolated as per our previous work (Ho et al., 2011). Over 150,000 reads from each sample were obtained. A majority of the reads (90%) were mapped to the coding regions (CDS) and known ncRNAs of the *E. coli* genome whereas 10% of the total reads were mapped onto the plasmid sequence (Table S3). Based on the read-mapping patterns, we identified six plasmid-encoded sRNAs that were all located at intergenic regions. Their details are summarized in Table 1. The sRNA sequences are presented in Table 2.

**Table 1:** General information of plasmid-encoded sRNAs identified in this study.

**Name**	**Region**	**Strand**	**5′-end**	**3′-end**	**Length**	**Adjacent genes**
NDM-sR1	Backbone	> > >	338	413	76	*repC*/*repB*
NDM-sR2	Backbone	> < >	462	399	64	*repC*/*repB*
NDM-sR3	Variable	> < >	29092	29017	76	Δ*tnpA*Tn*1*/*insL*
NDM-sR4	Variable	> < <	46777	46677	101	*D616_p59057*/*tnpA*
NDM-sR5a	Backbone	> > >	54935	54989	55	*D616_p59082/D616_p59083*
NDM-sR5b	Backbone	> > >	58418	58472	55	D616_p59086/D616_p59087
NDM-sR5c	Backbone	> > >	59721	59773	53	D616_p59087/D616_p59088
NDM-sR5d	Backbone	> > >	61983	62037	55	*D616_p59091*/*klcA*
NDM-sR6	Variable	< < <	8663	8343	321	*D616_p59071*/*aacC2*

**Table 2:** Sequence information of plasmid-encoded sRNAs identified in this study.

**Name**	**Sequence 5′-3′**
NDM-sR1	AUUUAGCACGGGAAGAGCGGCCCGGAGAUGUACCGCUUGGGGUGUGGCAGGAGCCACAAAAAGUAAAACCCCCUGA
NDM-sR2	GUUUGCGCCCCCUGAUUUCAUCUCUGACCGGCCAAAGUGAGAGGAUGAAUCAGGGGGUUUUACU
NDM-sR3	GUCAAUUGUUUAGGUGUUCCCGCCUCCAUCCGGGGCAAAACAUAAAAACCCGAUCUCACCAGUCGGGUUUUUUUGU
NDM-sR4	GAUUAACAAAUUAAUUUUCCGUUUACUCCGACAGAAAGUUAAGUAGUAAAAACGUCUAGUAAUUAAAAGGAGCCCUUCCUCACCAGUUGGGCUCCUUUUGU
NDM-sR5a	AUCGAAAGGCAAUCCGGUCUUUCGACGGUAGCGCCCCGACCGGCGCAGGAGUCAC
NDM-sR5b	AUCGAGAGGCAAUGAGGCUUUUCGCGGUAGCGCCCCGACUGGCGCAGGAGUAACA
NDM-sR5c	AUCGAAAGGCAAUGAGGCUUUUCGCGGUAGCGCCCCGACUGGCGCAGGAGUAA
NDM-sR5d	AUCGAAAGGCAAUCCGGUCUUUCGACGGUAGCGCCCCGACUGGCGCAGGAGUACA
NDM-sR6	AUUCUCGAUUGCUUUGCUAUCGAAGGAAAGCCGGAUGCGGUUGAAACUAUAGCAAAUGCUUACGUGAAGCUCGGUCGCCAUCGAGAAGGUGUCGUGGGCUUUGCUCAGUGCUACCUGUUCGACGCGCAGGACAUCGUGACGUUCGGCGUCACCUAUCUUGAGAAGCAUUUCGGAACCACUCCGAUCGUGCCUCCGCACGAGGCCGUCGAGCGCUCUUGCGAGCCUUCAGGUUAGAGGCCGUCGACAAUGAUAAUCUGGAUCAACGGACCUUUCGGCGCCGGAAAGACGACGCUCGCUAAGCGGCUGCGCGAUCGGCGUUCC

**Figure 1:**
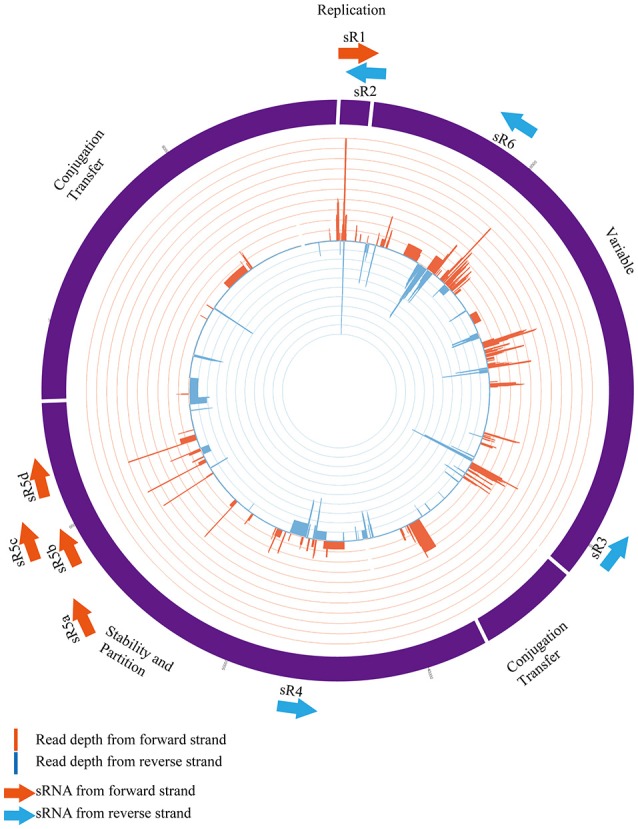
Distribution of six plasmid-encoded sRNAs in pNDM-HK plasmid. The sRNAs and their orientations are represented as arrows (orange and blue colours representing forward and reverse directions, respectively). NDM-sR1, sR2, sR4 and sR5 are located in the backbone region and NDM-sR3 and sR6 are located in the variable region. Strand-specific read depth is plotted within the inner circle at a log2 scale.

As depicted in Figure 1, these six plasmid-encoded sRNAs were distributed across different regions of the pNDM-HK plasmids, of which four (NDM-sR1, sR2, sR4, and sR5a-d) were located at the backbone region and two (NDM-sR3 and sR6) fell within the variable region. NDM-sR1 and sR2, which are located between *repC* and *repB* in the replication region, are two overlapping sRNAs with opposite orientations. They are 76 nt (NDM-sR1) and 64 nt (NDM-sR2) in length with 15 nt overlapping at the 3′-ends. Intriguingly, NDM-sR2 possessed the characteristic of the known counter-transcribed RNAs (ctRNA) because of the orientation (antisense RNA between *repC* and *repB*), sequence homology (Figure S1), and secondary structure (Athanasopoulos et al., 1995). As a negative post-transcriptional regulator of *repA*, ctRNA determines plasmid incompatibility and controls replication (Athanasopoulos et al., 1999; Izquierdo et al., 2005; Cervantes-Rivera et al., 2010). Unexpectedly, we identified a novel sRNA (NDM-sR1) that is antisense to this ctRNA, suggesting the possibility of a more intricate and complex replication control system in the pNDM-HK plasmid.

NDM-sR3 is a 76-nt sRNA located between Δ*tnpA*Tn*1* and *insL*, suggesting the association of transposable-element shuffling or dissemination between plasmids. NDM-sR4 is a 101-nt sRNA in the stability region and downstream to a transposase gene *tnpA* of IS26. For NDM-sR5, it is noteworthy that four highly similar copies were found and deemed NDM-sR5a-d. The sequence of NDM-sR5b and -sR5c has up to 96% identity. The occurrence of NDM-sR5 at several genomic locations with high sequence similarity indicates that duplication events might have taken place during the course of evolution. As these isoforms are all located at the stability region of the plasmid, they may play roles in plasmid partitioning or post-segregational stability. NDM-sR6 was detected at the 3′-UTR of *aacC2*, a gene conferring resistance to aminoglycoside. Intriguingly, 3′-UTRs was reported as a source of regulatory RNAs in bacteria (Gößringer and Hartmann, 2012), and we found three transcription start sites (TSSs) of NDM-sR6 (Figure S2). These results suggest that the *aacC2* gene may initiate other downstream consequences to the bacterial host through NDM-sR6. Whether NDM-sR6 is generated through internal processing of *aacC2* or is transcribed independently requires further investigation.

### Characterization of novel sRNAs from pNDM-HK

**Figure 2:**
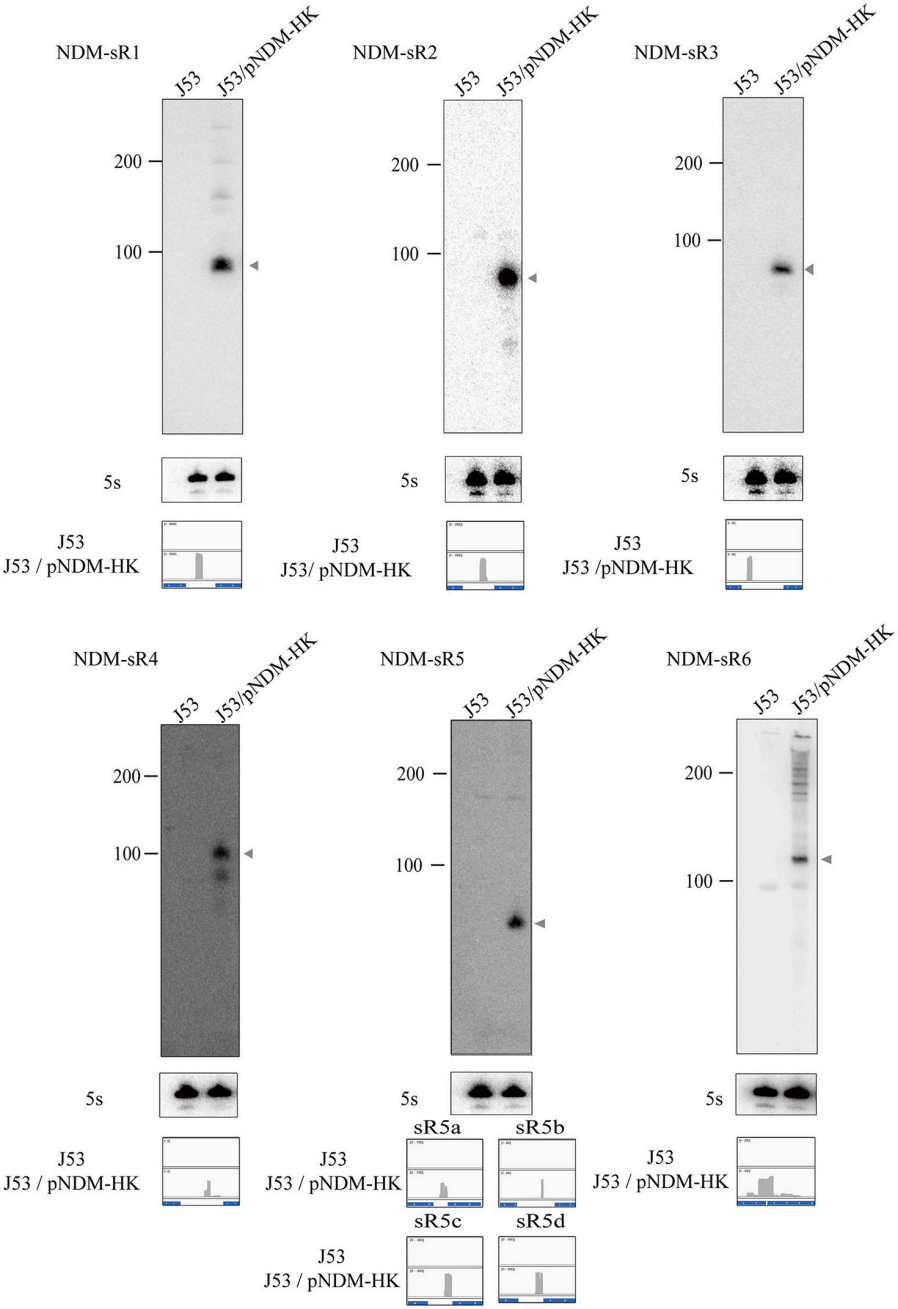
Northern blot analysis of plasmid-encoded sRNAs. The level of pNDM-HK encoded sRNAs, NDM-sR1 to sR6, was detected by radioactively-labelled sRNA-specific RNA probes. 5S rRNA was used as the loading control. Ladder sizes (nt) are labeled on the left, and each sRNA is indicated by an arrow on the right of the blot. The read counts of each sRNA was compared with the controls shown in IGV below the blot.

In order to validate and characterize the newly found intergenic sRNAs encoded from the pNDM-HK plasmid, we performed Northern blot analysis to identify their expression patterns. Total RNAs were extracted from transconjugants grown at stationary phase and separated by denaturing gel electrophoresis. The RNAs were then transferred to the membrane, hybridized with the sRNA-specific radioactive labeled RNA probes and analyzed by autoradiography. As portrayed in Figure 2, all plasmid-encoded sRNAs were detected in the transconjugant but not in the J53 control, indicating that these sRNAs were produced from the pNDM-HK plasmid. The sRNAs ranged between 50 and 150 nt, and all of them showed a single major band as indicated by the Northern blot. The original images of the Northern blots are found in Figure S3.

**Figure 3:**
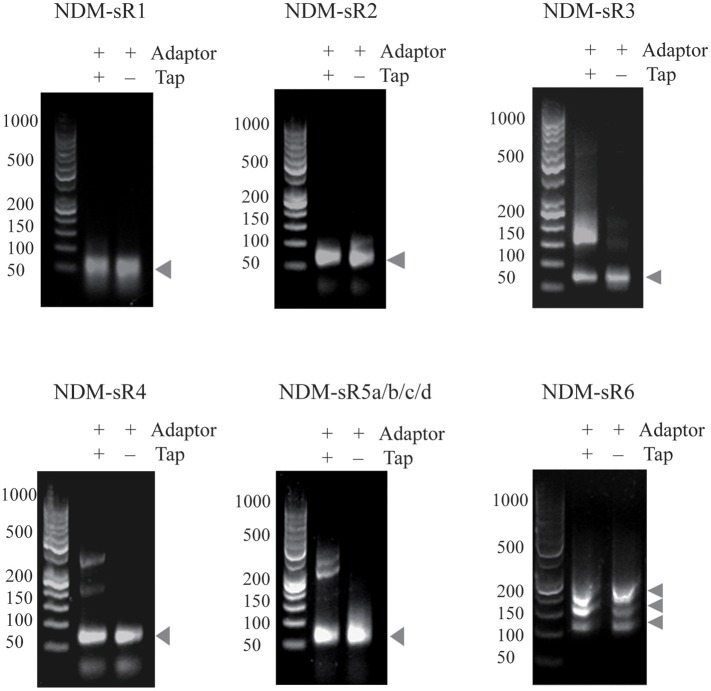
The 5′RACE analysis of sRNAs in pNDM-HK plasmid. Total RNAs were reverse-transcribed in the presence or absence of TAP, and PCR were performed with specific 5′RACE primers. The TSS of each sRNAs were identified on the basis of stronger signals in TAP-treated samples (as indicated by arrows).

**Figure 4:**
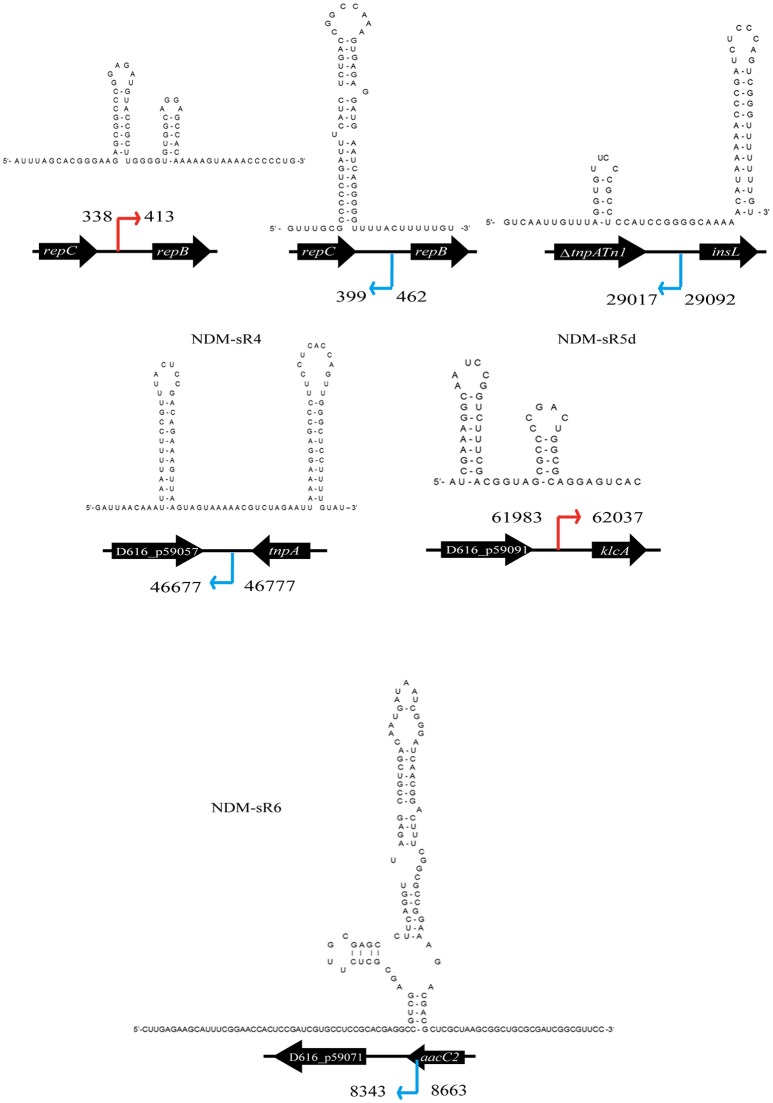
Secondary structure prediction of each sRNA from pNDM-HK. The secondary structure of each sRNA were predicted using RNAfold. The orientation and coordinates of the sRNAs are shown according to the TSS and sequencing results.

To identify the TSS of these six novel sRNAs, 5′-RACEs of each newly transcribed sRNA was performed. As shown in Figure 3, the TSS of each sRNA (indicated by arrows) was identified on the basis of a stronger signal in Tobacco Acid Pyrophosphatase (TAP)-treated samples. All of these sRNAs exhibited single TSS except NDM-sR6 that possessed three different TSSs (Figure S2). The calculated sizes of NDM-sR6 were 147, 250, and 321 nucleotides. The secondary structures of the sRNAs were also predicted with the RNAfold program (Gruber et al., 2008) and are depicted in Figure 4. Most contain at least one stem loop and a stretch of uridine sequences, which indicates the typical structure of bacterial sRNA.

### Phylogenetic analysis of plasmid-encoded sRNAs

**Figure 5:**
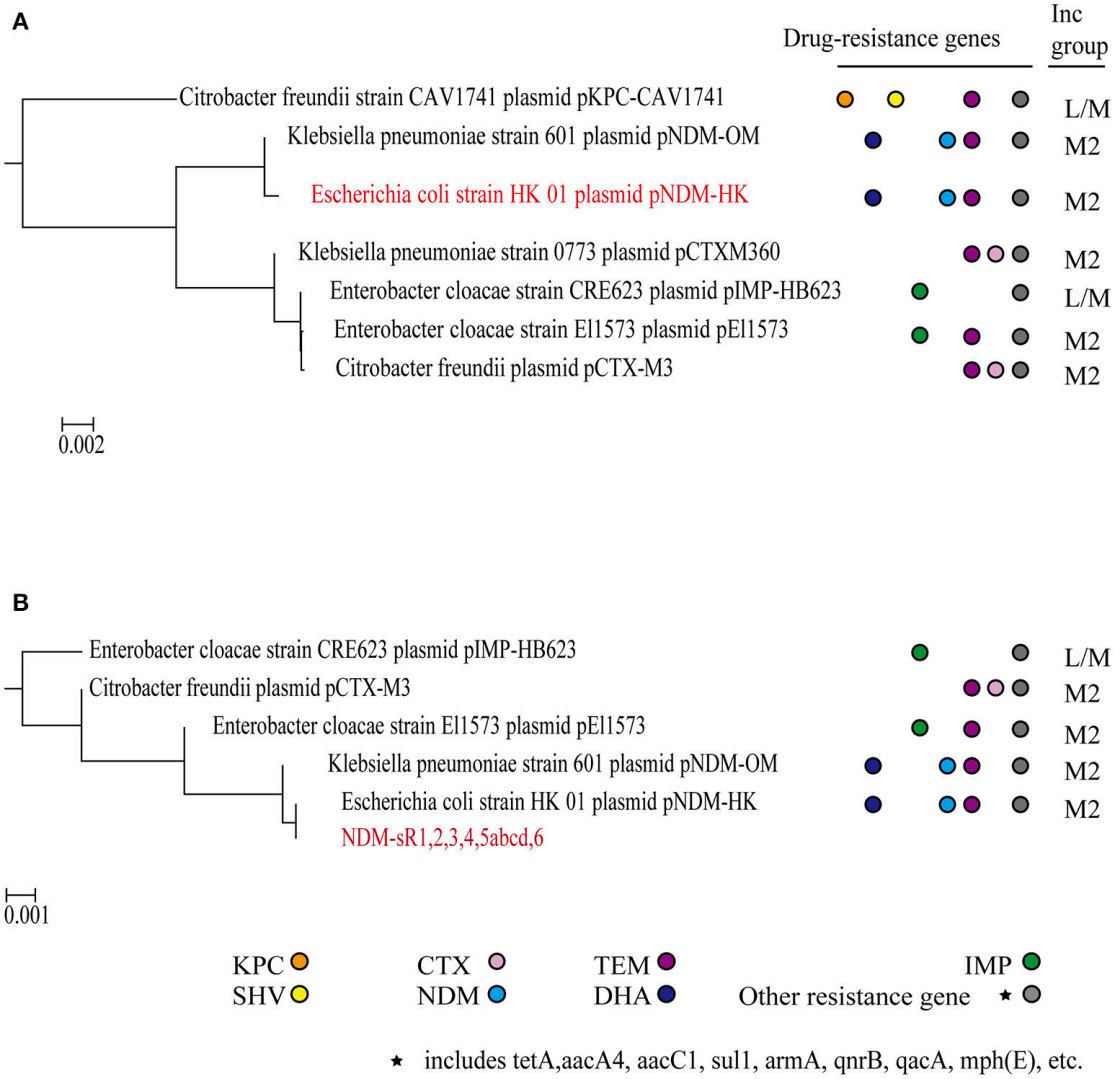
Phylogenetic analysis. Phylogenetic trees were built based on whole pNDM-HK plasmid sequences **(A)** and six plasmid-encoded sRNAs **(B)**. The drug resistance genes and incompatible groups of the plasmids are shown.

Phylogenetic profiling has been instrumental in inferring the evolutionary relationships between plasmids, especially the acquisition of genes by integration, transposition, and recombination. Phylogenetic trees are normally constructed from either the backbone sequence or the MDR regions of plasmids (Ma et al., 2007; Brilli et al., 2008; Norberg et al., 2011). In order to understand the contribution of plasmid-encoded sRNAs to the phylogenetic relationship of plasmids, we constructed and compared phylogenetic trees based on either the whole plasmid sequence or six novel sRNAs (refers to section Materials and Methods). As seen in Figure 5, the pNDM-HK and pNDM-OM plasmids are closely related phylogenetically, just as previously reported. Remarkably, we found that the plasmids in our sRNA-phylogenetic tree all belonged to the IncL/M group. Most of the resistance genes in pNDM-HK were acquired from these plasmids through transposition and recombination (Bonnin et al., 2013; Adamczuk et al., 2015). Plasmids such as pCTX-M3, pEl1573, and pNDM-OM were reported to be involved in the evolutionary pathway of pNDM-HK. Based on a recently published paper that re-designated IncL and IncM plasmids from the IncL/M group, most of the plasmids in our sRNA-phylogenetic tree were assigned to IncM2 (Carattoli et al., 2015) (pNDM-HK, pNDM-OM, pCTXM360, pEI1573, and pCTX-M3), except for plasmids without re-designation information (pIMP-HB623 and pKPC-CAV1741). We also constructed a phylogenetic tree based on either the backbone region of pNDM-HK or sRNAs at the backbone region, including NDM-sR1, 2, 4, and 5 (Figure S4). Although the resolution of the sRNA-phylogenetic tree is not as good as the tree constructed from the backbone region, we can still identify the plasmids closely related to pNDM-HK, such as pCTX-M360, pCTX-M3, pEl1573, and pNDM-OM. These results strongly suggest that plasmid-encoded sRNAs can be used for phylogenetic analysis to reveal evolutionary relationships in other MDR plasmids.

### Hfq dependency of NDM-sR3

**Figure 6:**
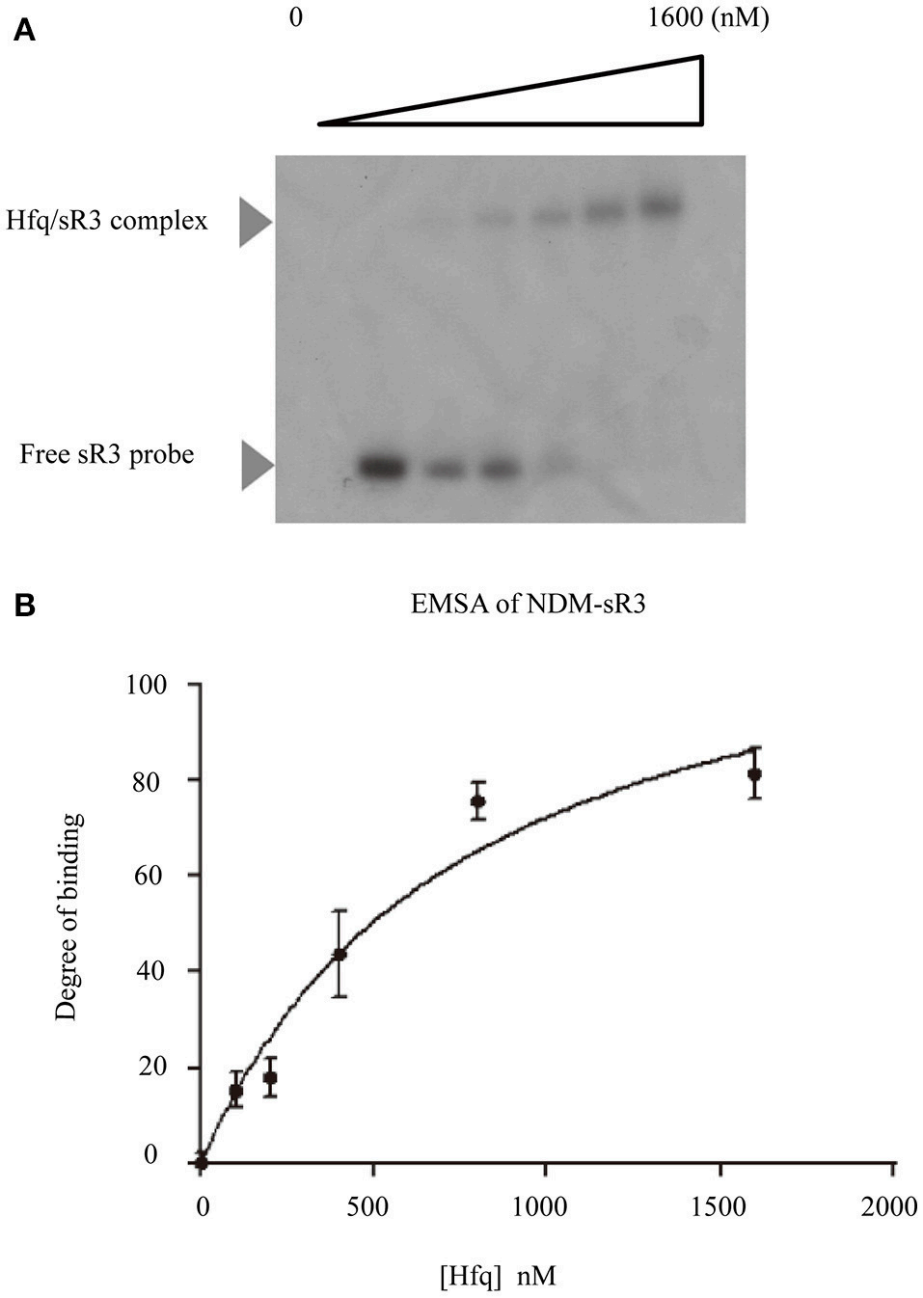
Electrophoretic mobility shift assay and determination of dissociation constant of NDM-sR3. **(A)** Electrophoretic mobility shift assay of ^32^P-labeled NDM-sR3 sRNA and purified *E. coli* His-tagged Hfq protein. Free sR3 sRNA and Hfq/sR3 complex are indicated by arrows. **(B)** Binding isotherm of the NDM-sR3/Hfq complex. The curve was fit using SigmaPlot and the dissociation constant of NDM-sR3 was calculated.

As illustrated in Figure 4, NDM-sR3 contains a polyU stretch at the 3′-end stem-loop and this stretch was commonly considered an Hfq-binding sequence (Otaka et al., 2011). As Hfq is the RNA chaperone that plays a significant role in sRNA biogenesis and function, we went on to assess if it interacts with NDM-sR3. We performed an Electrophoretic Mobility Shift Assay (EMSA) using *in-vitro* transcribed and radioactive-labeled NDM-sR3 RNA with purified recombinant Hfq protein. In Figure 6A, the NDM-sR3-Hfq complex was gradually formed and shifted in the gel as the concentration of Hfq increased. To quantitate the dissociation constant (K_*d*_), the binding curve was plotted against an increasing concentration of Hfq based on the intensity of the bands (Figure 6B). The K_*d*_ of NDM-sR3 to Hfq was calculated as 750 nM. This suggests that NDM-sR3 interacts with Hfq at medium affinity compared with other sRNAs, of which the K_*d*_ are normally <100 nM (Henderson et al., 2013). We also tested the affinity between Hfq and another plasmid-encoded sRNA, NDM-sR2, as a control. Nearly no sR2-protein complex formed in the presence of 1,600 nM Hfq protein, indicating there was no interaction between Hfq and NDM-sR2 (Figure S5).

### Stability of NDM-sR3 *in vivo*

**Figure 7:**
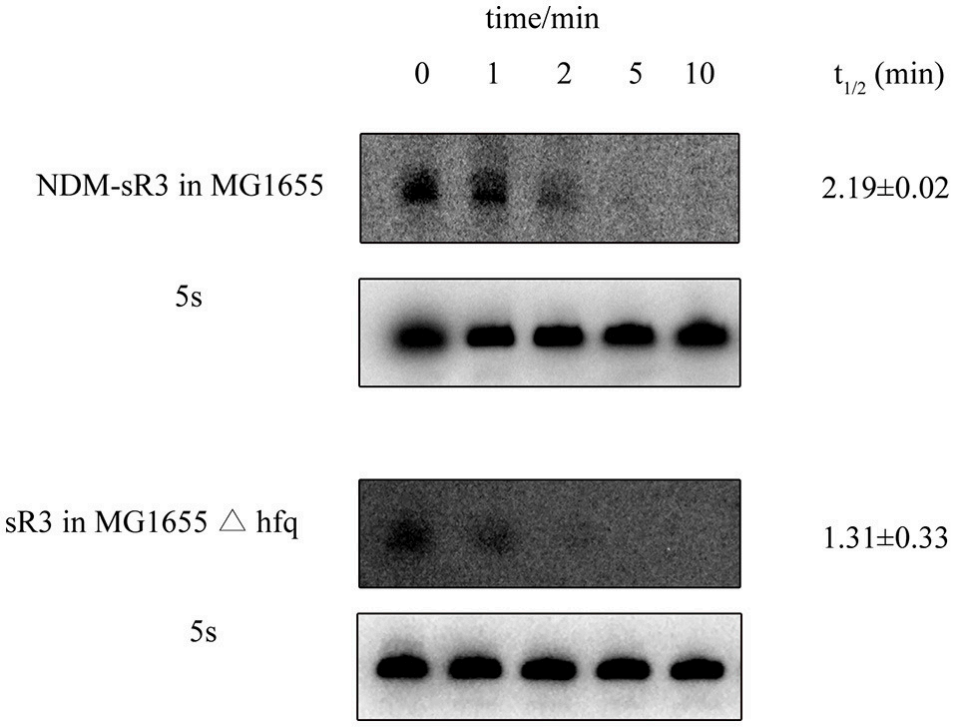
Stability of NDM-sR3 determined by *in vivo* degradation. MG1655 and MG1655Δ*hfq* carrying pACYC184-sR3 were grown to mid-log phase, and cells were harvested at the indicated time points after termination of RNA synthesis by rifampicin. RNA levels of sR3 were probed by a radioactively-labeled sR3-specific probe while 5S was used as a control. The intensity of each band was quantitated by ImageJ and normalized by the intensity of 5S. The half-life was calculated as described in the Materials and Methods.

Hfq could protect the sRNA from ribonuclease degradation and increase its stability in bacteria. After showing that Hfq interacts with NDM-sR3, we further examined whether Hfq can protect NDM-sR3 in the bacterial host. The abundance of NDM-sR3 was monitored by Northern blot analysis in the presence (MG1655) and absence (MG1655Δ*hfq*) of Hfq after the addition of rifampicin, a bacterial DNA-dependent RNA synthesis inhibitor. The results showed that NDM-sR3 was rapidly degraded in both strains, and it completely disappeared in 10 min after rifampicin treatment (Figure 7). The half-life of NDM-sR3 in MG1655 was determined as 2.19 ± 0.02 min, whereas that in MG1655Δ*hfq* was 1.31 ± 0.33 min, indicative of NDM-sR3 being stabilized and protected by Hfq. The short half-life of NDM-sR3 is consistent with previously reported plasmid-encoded antisense RNA (RNAI) (Wagner and Brantl, 1998).

### Regulatory roles of NDM-sR3 in bacteria

**Figure 8:**
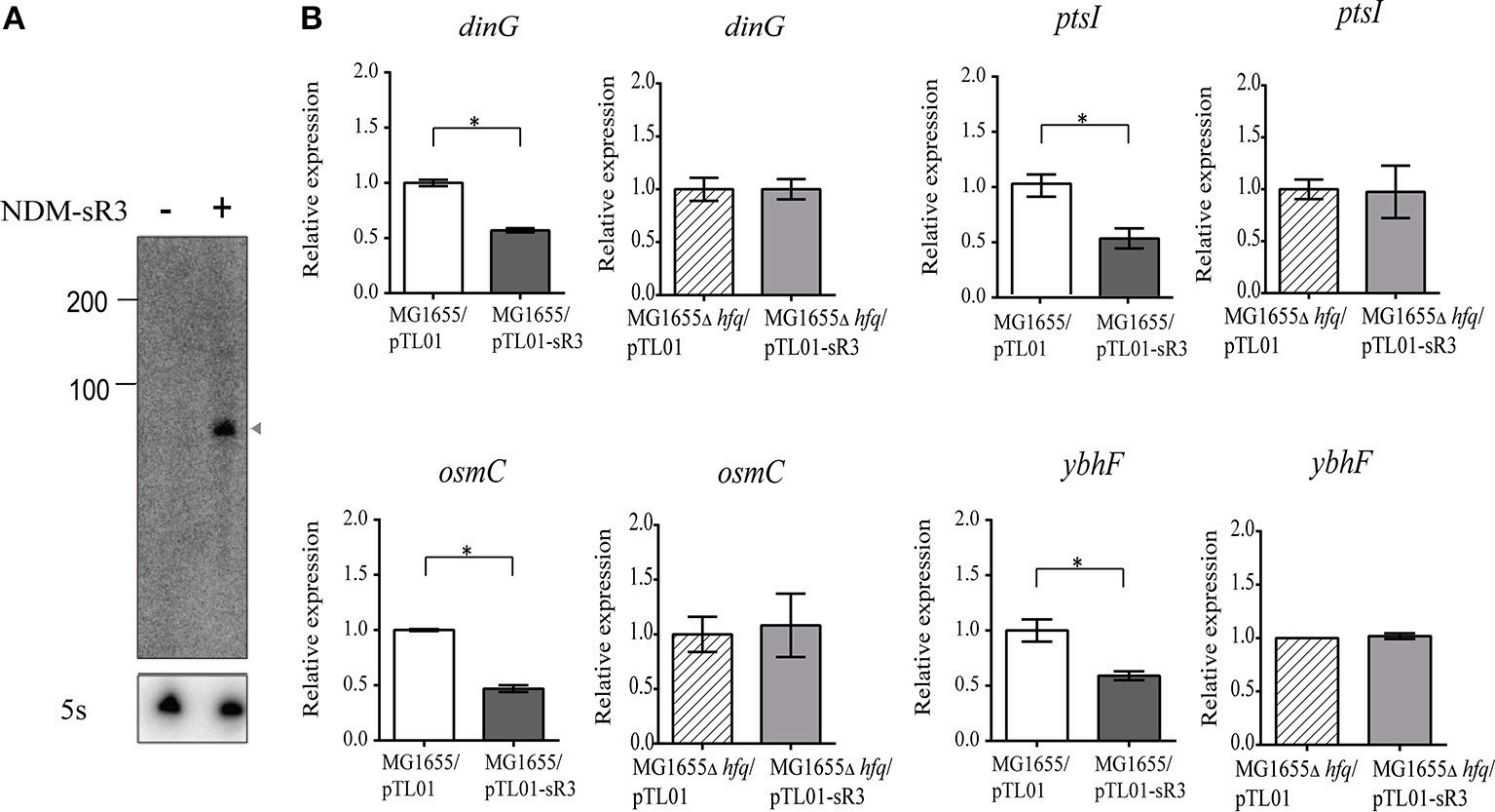
Target genes regulation of NDM-sR3 in *E. coli*. NDM-sR3 was overexpressed in *E. coli* (MG1655) and the expression levels of target genes were measured by qRT-PCR. **(A)** Northern blot analysis of NDM-sR3 in MG1655. **(B)** Measurement of the transcript levels of NDM-sR3's target genes by qRT-PCR. The relative mRNA expression levels are shown and the results are expressed as the means of at least three independent experiments. The statistical significance of the comparison: ^*^p < 0.05, ^**^p < 0.01, ^***^p < 0.001.

We have identified six pNDM-HK-encoded sRNAs, and they are constitutively expressed within the bacterial host. In order to further characterize the functional roles of sRNAs in the bacterial host, we selected NDM-sR3 that is located at the variable region and associated with IS26-mediated insertions in pCTX-M3 for further investigation. To determine the regulatory role of NDM-sR3 in the gene expression of bacteria, we cloned the TU of NDM-sR3 into the pTL01 plasmid and transformed it into the model strain of *E. coli* MG1655. We utilized a target gene prediction program, IntaRNA (Busch et al., 2008; Wright et al., 2014), to predict target genes on the bacterial host chromosome and validated their expression level by qRT-PCR in the NDM-sR3 overexpressed bacteria. As shown in Figure 8A, NDM-sR3 was successfully expressed and detected according to Northern blot analysis. Moreover, we found that overexpression of NDM-sR3 down-regulated the expression level of *dinG, osmC, ptsI*, and *ybhF* by approximately 50% (Figure 8B). *dinG* is an ATP-dependent helicase that confers DNA helicase activity in terms of DNA repair and replication. *osmC* is an osmotically inducible, stress-inducible membrane protein as well as peroxiredoxin (Cussiol et al., 2010; Cheng et al., 2012). *ptsI* is a cytoplasmic protein component of the phosphoenolpyruvate:sugar phosphotransferase system (Ginsburg and Peterkofsky, 2002). Finally, *ybhF* is predicted as part of the ATP-dependent efflux pump (Zhang et al., 2007).

As we have shown that NDM-sR3 is stabilized by Hfq protein in *E. coli*, we continued to evaluate whether the regulatory effects of NDM-sR3 on its target genes are also Hfq-dependent. We transformed NDM-sR3 into *E. coli* MG1655Δ*hfq* and measured the expression levels of the target genes. Of note, none of the genes exhibited any influence in a regulatory sense in the absence of Hfq, indicating that the regulatory role of NDM-sR3 is Hfq-dependent.

## Discussion

Small RNA sequencing has undoubtedly revolutionized the discovery of bacterial sRNAs at high resolution and with precise accuracy in recent years (Livny and Waldor, 2007). Currently more than 80 sRNAs have been identified in the *E. coli* genome. These sRNAs regulate genes in response to external stimuli as well as various essential processes in bacterial physiology (Raghavan et al., 2011). Nevertheless, the identification and understanding of MDR plasmid-encoded sRNAs in the context of dissemination, fitness and conferral of drug resistance with regards to the bacterial host is very limited. The plasmid we studied, pNDM-HK, belongs to the broad host range IncL/M incompatibility group and spreads in *Enterobacteriaceae* and Gram-negative non-fermenters, such as *E. coli, K. pneumoniae, E. cloacae*, and *C. freundii* (Ho et al., 2011). It is reported that the backbone region (~55 kb) of pNDM-HK shared 97% identity to pEL60 from a plant pathogen, *E. amylovora*, whilst the major variable region (28.9 kb) showed extensive homology to pCTX-M3 in *C. freundii*, pMUR050 in *E. coli* and pKP048 in *K. pneumoniae* (Ho et al., 2011). In our study, we identified six novel sRNAs encoded from the pNDM-HK plasmid and performed functional studies with them using bioinformatics and biochemical analysis. These sRNAs are highly conserved in four other plasmids found in clinical isolates, including pCTX-M3, pIMP-HB623, pEI1573, and pNDM-OM, suggesting their functional significance among these plasmids. Of particular interest, the phylogenetic tree constructed from these sRNAs showed the evolutionary pathway of pNDM-HK plasmid and the possible order of resistance gene acquisition and insertion (Figure 5). Therefore, we propose a new way to construct a phylogenetic tree employing plasmid-encoded sRNAs to study the dissemination of emerging MDR plasmids. The classification of IncL/M plasmids has been evolving and a recently published paper has re-designated the incompatible group IncL/M into IncL, IncM1 and IncM2 (Carattoli et al., 2015). Surprisingly, our sRNA-phylogenetic tree clustered IncM2 plasmids from other IncL/M sub-groups, suggesting an additional and alternative approach to distinguish different types of incompatible groups using sRNAs (Figure 5). These phylogenetic trees could provide information in the plasmid evolutionary pathway contributed by gene acquisition from relevant plasmids. An obvious advantage of this method is that novel plasmids could be classified without complete sequences, which enables a quick screening for diagnosis.

An extensive blast search against the NCBI nr/nt database (Altschul et al., 1997; Tatusova and Madden, 1999) using the six sRNA sequences show that all of them can be found in bacterial plasmids. Notably, four of the six sRNAs that are located at the backbone region of pNDM-HK (NDM-sR1, sR2, sR4, and sR5) were also found in pEL60. pEL60 possesses a typical IncL/M backbone without a resistance gene or insertion sequence, and is believed to evolve into pNDM-HK, pCTX-M3, and pNDM-OM by transposon integration and resistance gene acquisition (Bonnin et al., 2013). NDM-sR3 and sR6, on the other hand, are possibly associated with drug resistance genes according to the blast results.

In bacteria, sRNAs are the most abundant class of post-transcriptional regulators. The plasmid-encoded sRNAs have been reported to control plasmid replication, conjugation and maintenance. In our study, we identified NDM-sR2 as a ctRNA, an antisense RNA that is found in the *repABC* plasmids. ctRNA is a strong trans-incompatibility factor that modulates the expression level of *repA*. It is worth highlighting that our data also identified a novel sRNA, NDM-sR1, in the *repABC* region, which is in the same orientation as *repABC* but antisense to the ctRNA (NDM-sR2). These two sRNAs are highly conserved in IncL/M plasmids. These newly found sRNAs raise the possibility of additional attenuation and control mechanisms pertaining to the replication of pNDM-HK as well as all IncL/M plasmids. One possible mechanism is that NDM-sR1 sequesters NDM-sR2 and represses pseudoknot formation as well as *repA* expression. However, the interaction between NDM-sR1 and -sR2 and their regulation and modulation of *repA* expression as well as plasmid replication is still unclear and further study is required.

In the functional study of NDM-sR3, we found that the chromosomal target genes (*dinG, osmC, ptsI*, and *ybhF*) were down-regulated under the overexpression of NDM-sR3. Intriguingly, *dinG* is the DNA damage-inducible member of the helicases and was found to promote replication across highly transcribed regions in the *E. coli* genome (Boubakri et al., 2010). Down-regulation of *dinG* in the presence of plasmid-encoded NDM-sR3 will presumably reduce the replication of the host genome and may enhance the fitness of the plasmid. Indeed, these target genes are highly conserved among common bacterial hosts found at hospitals, such as *K. pneumonia* and *E. cloacae*. Future molecular studies should focus on elucidating the functional roles of these down-regulated genes in the context of plasmid fitness as well as drug resistance. A generic interaction could exist between plasmid-encoded sRNAs and the host genome.

## Author contributions

H-KK, WL, TC, PH, and TL: Conceived and designed the experiments; H-KK, and QP: Performed the experiments; XL and CL: Analyzed the data; H-KK, XL, QP, and TL: Wrote the paper.

### Conflict of interest statement

The authors declare that the research was conducted in the absence of any commercial or financial relationships that could be construed as a potential conflict of interest.

## Supplementary material

Figure S1 Sequence homology of NDM-sR2 to known counter-transcribed RNA.

Figure S2 Three different transcription start sites identified in NDM-sR6.

Figure S3 Original image of Northern blot analysis.

Figure S4 Phylogenetic analysis of pNDM-HK using backbone information. **(A)** Phylogenetic tree constructed on backbone region of pNDM-HK. **(B)** Phylogenetic tree built on sRNAs encoded in backbone region of pNDM-HK (NDM-sR1, 2, 4, and 5).

Figure S5 Electrophoretic mobility shift assay of NDM-sR2. Electrophoretic mobility shift assay of ^32^P-labeled NDM-sR2 sRNA and purified *E. coli* His-tagged Hfq protein. Free sR2 sRNA and Hfq/sR2 complex are indicated by arrows.

Table S1 *E. coli* strains and plasmids used in this study.

Table S2 Primers used in this study.

Table S3 Mapping statistics of sequencing data.
