# Supplementary figures and images for: Identification of Plasmid-Encoded sRNAs in a blaNDM-1-Harboring Multidrug-Resistance Plasmid pNDM-HK in Enterobacteriaceae
Source: Front Microbiol. 2018 Mar 27;9:532. doi: 10.3389/fmicb.2018.00532 (PMC5880898; doi:10.3389/fmicb.2018.00532)

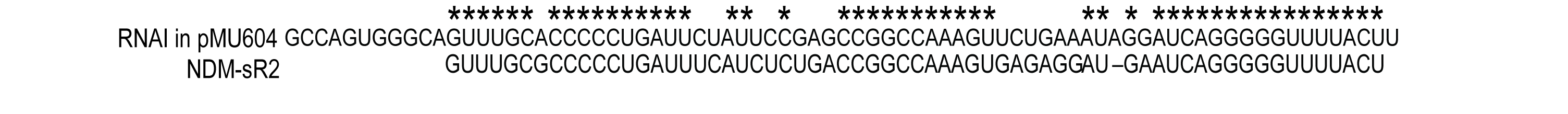

Supplement: Figure S1 — Sequence homology of NDM-sR2 to known counter-transcribed RNA. [file Image1.TIF]

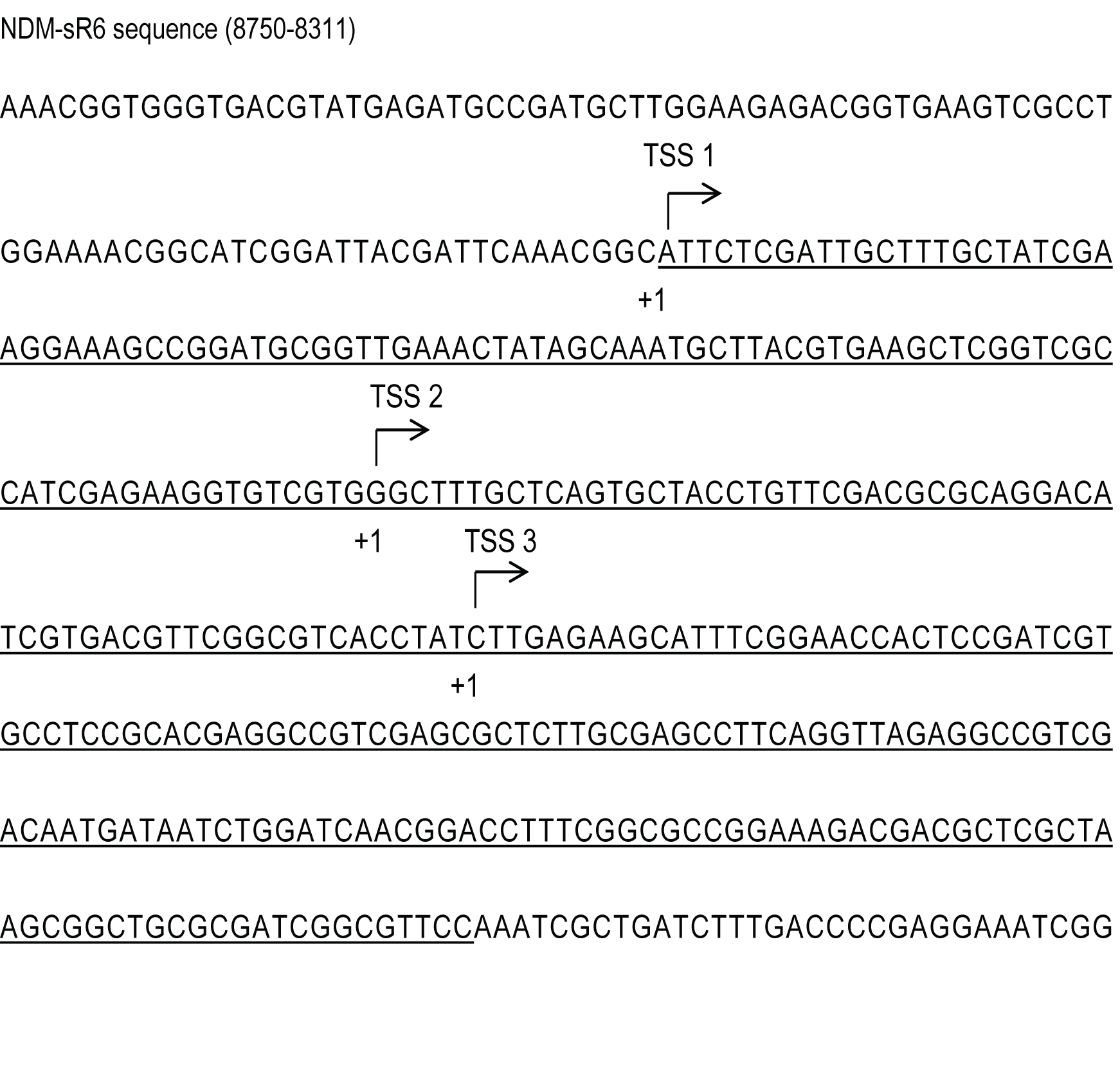

Supplement: Figure S2 — Three different transcription start sites identified in NDM-sR6. [file Image2.TIF]

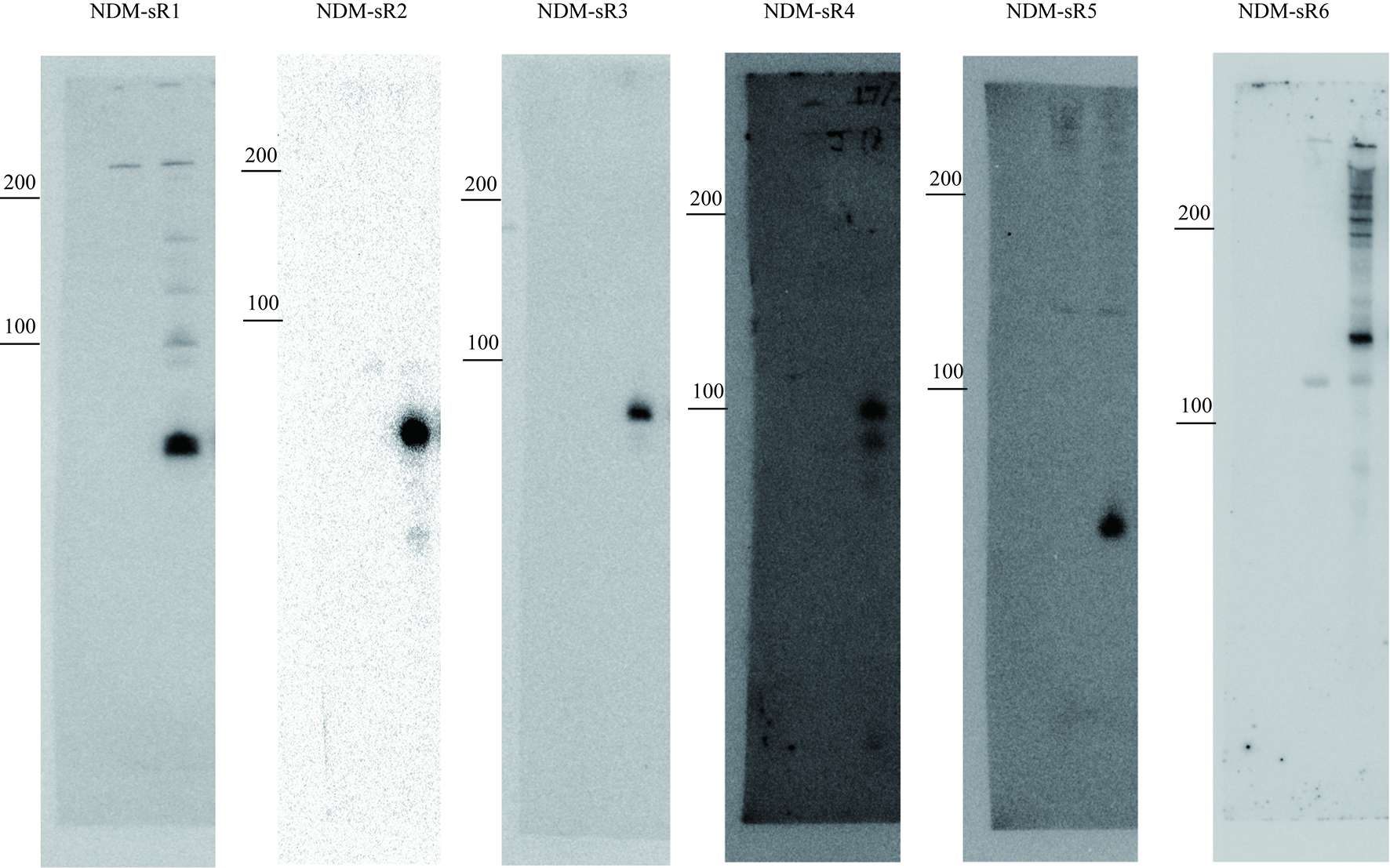

Supplement: Figure S3 — Original image of Northern blot analysis. [file Image3.tif]

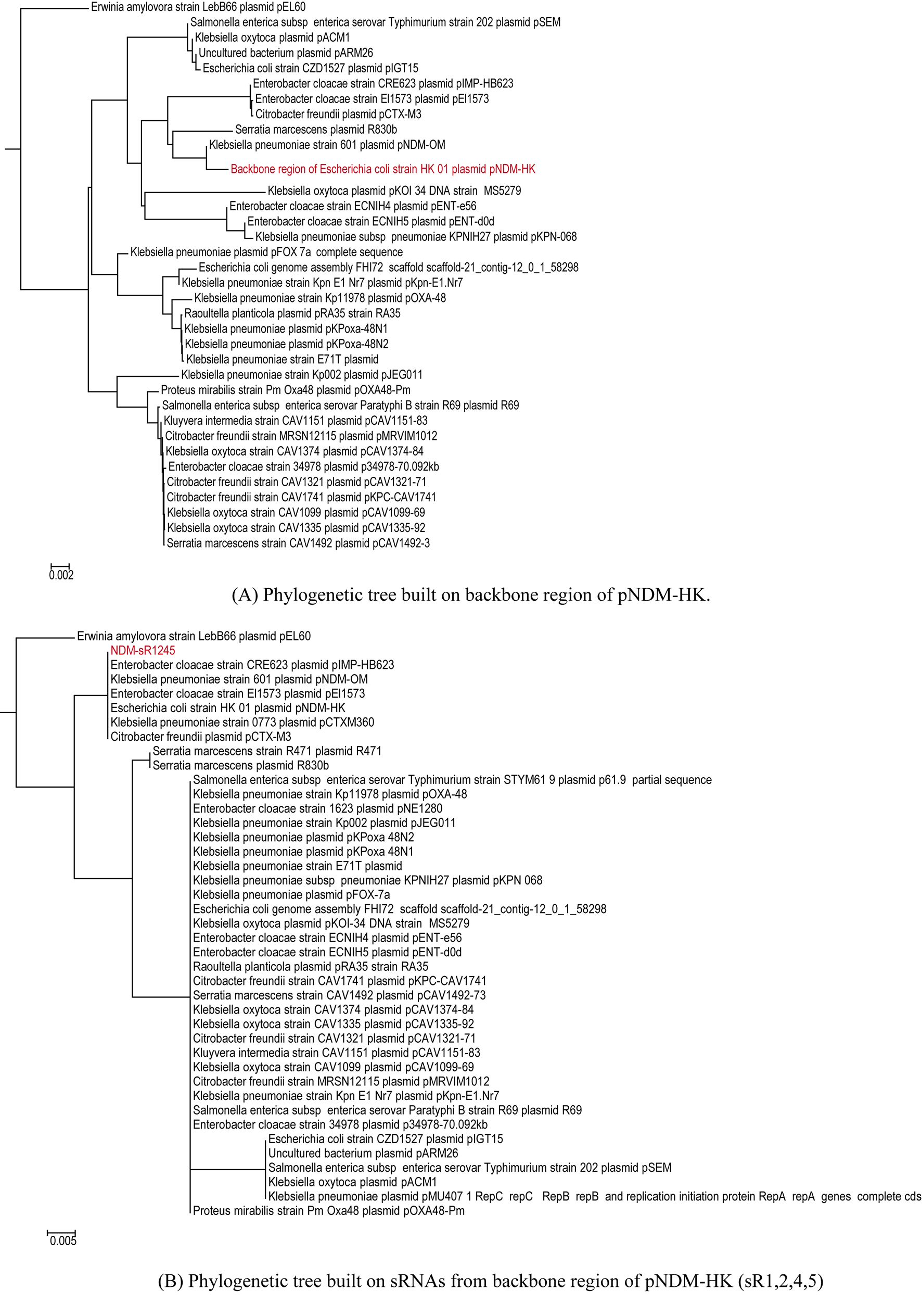

Supplement: Figure S4 — Phylogenetic analysis of pNDM-HK using backbone information. (A) Phylogenetic tree constructed on backbone region of pNDM-HK. (B) Phylogenetic tree built on sRNAs encoded in backbone region of pNDM-HK (NDM-sR1, 2, 4, and 5). [file Image4.tif]

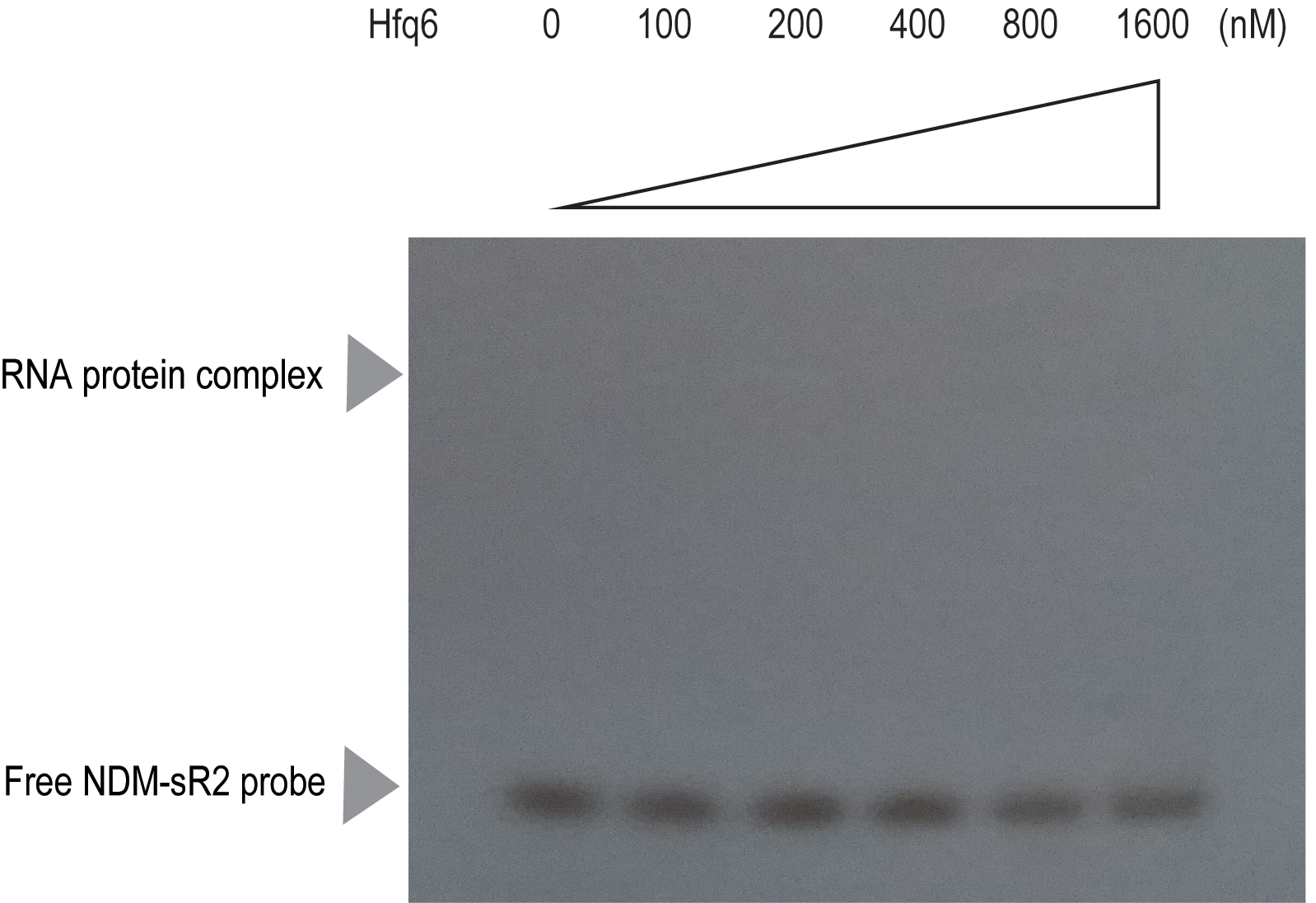

Supplement: Figure S5 — Electrophoretic mobility shift assay of NDM-sR2. Electrophoretic mobility shift assay of 32P-labeled NDM-sR2 sRNA and purified E. coli His-tagged Hfq protein. Free sR2 sRNA and Hfq/sR2 complex are indicated by arrows. [file Image5.TIF]
