# Supplementary material for: Identification of Plasmid-Encoded sRNAs in a blaNDM-1-Harboring Multidrug-Resistance Plasmid pNDM-HK in Enterobacteriaceae
Source: Front Microbiol. 2018 Mar 27;9:532. doi: 10.3389/fmicb.2018.00532 (PMC5880898; doi:10.3389/fmicb.2018.00532)
Supplement: Table S1 — E. coli strains and plasmids used in this study. [file Table1.DOCX]

**Table S1. *E. coli* strains and plasmids used in this study.**

| Construct | Relevant genotype | Ref or Source |
| --- | --- | --- |
| **Strains** |  |  |
| J53 | F ^−^ met pro Azi^r^ | ([Yi et al., 2012](#_ENREF_2)) |
| DH5α | F ^−^ φ80d*lacZΔM15 Δ*(*lacZYA-argF*)*U169 deoR*  *recA1 endA1 hsdR17*(*r_k_- m_k_+*) *phoA supE44 λ-thi-1 gyrA96 relA* | This lab |
| BL21(DE3)pLysS | F ^−^ *ompT gal dcm lon hsdSB(r_B_- m_B_-*) λ(DE3) pLysS, Cm^r^ | This lab |
| MG1655 | F ^−^ λ ^−^ *rph*-1 | This lab |
|  |  |  |
| **Plasmids** |  |  |
| pNDM-HK | pEL60 backbone with three DNA insertions, Amp^r^ | ([Ho et al., 2011](#_ENREF_1)) |
| pACYC184 | Cm^r^, Tet^r^ | NEB |
| pET28a | phage T7 promoter, Kn^r^ | Novagen |
| pTL01 | pACYC184 with *Xho* I, 2823 CCAATC to CTCGAG, Cm^r^ | This study |
| pTL02 | pACYC184 with Transcription unit of NDM-sR3 in *Xho* I, Cm^r^ | This study |

**References**

Ho, P.L., Lo, W.U., Yeung, M.K., Lin, C.H., Chow, K.H., Ang, I., et al. (2011). Complete sequencing of pNDM-HK encoding NDM-1 carbapenemase from a multidrug-resistant Escherichia coli strain isolated in Hong Kong. *PLoS One* 6(3)**,** e17989. doi: 10.1371/journal.pone.0017989.

Yi, H., Cho, Y.-J., Yong, D., and Chun, J. (2012). Genome sequence of Escherichia coli J53, a reference strain for genetic studies. *Journal of bacteriology* 194(14)**,** 3742-3743.
