# Supplementary material for: Identification of Plasmid-Encoded sRNAs in a blaNDM-1-Harboring Multidrug-Resistance Plasmid pNDM-HK in Enterobacteriaceae
Source: Front Microbiol. 2018 Mar 27;9:532. doi: 10.3389/fmicb.2018.00532 (PMC5880898; doi:10.3389/fmicb.2018.00532)
Supplement: Table S2 — Primers used in this study. [file Table2.DOCX]

**Table S2. Primers used in this study.**

| Name | Seq 5’-3’ |
| --- | --- |
| NB-probe NDM-sR1 | TGG CTC CTG CCA CAC CCC AAG CGG TAC ATC TC |
| NB-probe NDM-sR2 | GAT TCA TCC TCT CAC TTT GGC CGG TCA GAG ATG |
| NB-probe NDM-sR3 | CGG ATG GAG GCG GGA ACA CCT AAA CAA TTG AC |
| NB-probe NDM-sR4 | GCC CAA CTG GTG AGG AAG GGC TCC TTT TAA TTA CTA GAC G |
| NB-probe NDM-sR5a | GTC GGG GCG CTA CCG TCG AAA GAC CGG ATT GCC T |
| NB-probe NDM-sR5b/5c | CTG CGC CAG TCG GGG CGC TAC CGC GAA AAG CCT CAT T |
| NB-probe NDM-sR5d | CTG CGC CAG TCG GGG CGC TAC CGT CGA AAG ACC GGA TT |
| NB-probe NDM-sR6-F | AAT TTA ATA CGA CTC ACT ATA GGG AAC GCC GAT CGC GCA GCC GC |
| NB-probe NDM-sR6-R | ACC ACT CCG ATC GTG CCT CCG CAC GAG |
| Probe 5S rRNA | TAC TCT CGC ATG GGG AGA CCC CAC ACT ACC ATC GG |
| T7-sR3-F | AAT TTA ATA CGA CTC ACT ATA GGG TCA ATT GTT TAG GTG TTC CCG |
| T7-sR3-R | CTG CCA GAA GGG CGG CAC |
| Hfq-F | CTA CCA TGG CTA AGG GGC AAT CTT TAC |
| Hfq-R | GTT CTC GAG TCA GTG GTG GTG GTG GTG GTG AAG CTT TTC GGT TTC TTC GCT GTC CTG TTG CG |
| XhoI-sR3-F | GAT ATA CTC GAG CCC TAT TCC GGG TGG TTT GC |
| XhoI-sR3-R | GAT ATA CTC GAG CTG CCA GAA GGG CGG CAC |
| 5’RACE-sR1-R1 | GTA CAT CTC CGG GCC GCT CTT |
| 5’RACE-sR1-R2 | GCC ACA CCC CAA GCG GTA CAT CTC |
| 5’RACE-sR2-R1 | CCG GTC AGA GAT GAA ATC AGG |
| 5’RACE-sR2-R2 | ATC CTC TCA CTT TGG CCG GTC AGA |
| 5’RACE-sR3-R1 | CGG ATG GAG GCG GGA ACA CC |
| 5’RACE-sR3-R2 | GGC GGG AAC ACC TAA ACA ATT |
| 5’RACE-sR4-R1 | GC TCC TTT TAA TTA CTA GAC G |
| 5’RACE-sR4-R2 | CAA CTG GTG AGG AAG GGC TCC TTT |
| 5’RACE-sR5a-R1 | CC GGA TTG CCT TTC GAT GTG |
| 5’RACE-sR5a-R2 | CGC TAC CGT CGA AAG ACC GGA TTG |
| 5’RACE-sR6-R1 | CGG CCT CTA ACC TGA AGG CTC GCA AGA |
| 5’RACE-sR6-R2 | CTC GCA AGA GCG CTC GAC GGC CTC GTG CGG |
| qPCR-dinG-F | GAG AAG AAG GGC GAC ATC TG |
| qPCR-dinG-R | GCG GTA CTC ACC ACC AGC GT |
| qPCR-osmC-F | GGG AAC AGT ATC CAC CGA GAG |
| qPCR-osmC-R | GCA TTA ATG AAA GCG CCA TTG |
| qPCR-ptsI-F | CAA CAT CCT GGG CCT GAA GAT |
| qPCR-ptsI-R | GTG ATG AAA CCC AGC ACC TTC |
| qPCR-ybhF-F | TGG GGA TGG ATC TGA AAG AG |
| qPCR-ybhF-R | AAT TCT GTT CGA CCG TCA GG |
| qPCR-gapA-F | GCT CGT AAA CAC ATC ACC GC |
| qPCR-gapA-R | CGA TGT CCT GGC CAG CAT AT |

(Table S2 concluded)
