# Supplementary material for: Identification of Plasmid-Encoded sRNAs in a blaNDM-1-Harboring Multidrug-Resistance Plasmid pNDM-HK in Enterobacteriaceae
Source: Front Microbiol. 2018 Mar 27;9:532. doi: 10.3389/fmicb.2018.00532 (PMC5880898; doi:10.3389/fmicb.2018.00532)
Supplement: Table S3 — Mapping statistics of sequencing data. [file Table3.DOCX]

| **Reference genome** | **Type** | **Number of reads**  **(J53)** | **Number of reads**  **(J53/pNDM-HK)** |
| --- | --- | --- | --- |
| Chromosomal | CDS | 112,652 | 73,541 |
|  | known ncRNA | 169,701 | 71,540 |
|  | tmRNA | 708 | 540 |
|  | novel intergenic | 56,388 | 22,103 |
|  | novel antisense | 14,987 | 4,368 |
| Plasmid  pNDM-HK | novel intergenic | 6 | 9,967 |
|  | novel antisense | 0 | 112 |

**Table S3. Mapping statistics of sequencing data.**
